# Social Epigenetics and Equality of Opportunity

**DOI:** 10.1093/phe/pht019

**Published:** 2013-07-15

**Authors:** Michele Loi, Lorenzo Del Savio, Elia Stupka

**Affiliations:** Center for Translational Genomics and Bioinformatics, CEHUM, Universidade Do Minho and San Raffaele Scientific Institute; Istituto Europeo di Oncologia; Center for Translational Genomics and Bioinformatics, San Raffaele Scientific Institute

## Abstract

Recent epidemiological reports of associations between socioeconomic status and epigenetic markers that predict vulnerability to diseases are bringing to light substantial biological effects of social inequalities. Here, we start the discussion of the moral consequences of these findings. We firstly highlight their explanatory importance in the context of the research program on the Developmental Origins of Health and Disease (DOHaD) and the social determinants of health. In the second section, we review some theories of the moral status of health inequalities. Rather than a complete outline of the debate, we single out those theories that rest on the principle of equality of opportunity and analyze the consequences of DOHaD and epigenetics for these particular conceptions of justice. We argue that DOHaD and epigenetics reshape the conceptual distinction between natural and acquired traits on which these theories rely and might provide important policy tools to tackle unjust distributions of health.

## Introduction

In this article, we assess the implications for justice and health of the developmental origins of health and disease (DOHaD) hypothesis and findings in epigenetics that corroborate it. Other articles have addressed the ethical and social consequences of epigenetics (Rothstein *et al.,* 2009; Drake and Liu, 2010; Hedlund, 2012), arguing that the fight against epigenetically transmitted forms of disadvantage is more a political than an individual responsibility. In this essay, we discuss the scientific evidence on which this claim is based and defend a case for public health interventions based on epigenetic discoveries in a framework of equality of opportunity. Because the concept of equality of opportunity is interpreted in different ways by different moral traditions, we analyze the luck-egalitarian and the Rawlsian versions showing that the two overlaps significantly in this context.

Recent discoveries in epigenetics improve our understanding of how individual health and therefore opportunity is affected by early developmental events and previous generations’ environmental circumstances. As we explain at length below, epigenetics is defined as the study of the inheritance (between cells and/or organisms) of traits (gene expression or phenotypes) without changes to the underlying DNA sequence. As we shall indicate, there are three features of epigenetic traits that make them important for a *normative* analysis of health inequalities:

- Sensitivity to social structures: Some epigenetic phenomena are highly responsive to environmental changes, which are affected by social institutions.
- Early programing: Several epigenetic traits are established early-on in development, and their effects on health unfold throughout the life course.
- Trans-generational transmission: There is evidence in both animal models and epidemiological studies that epigenetic traits can be trans-generationally inherited. In addition to genetic inheritance that provides adaptive flexibility in the long (evolutionary) time span, epigenetics constitutes other, semi-stable, biological mechanisms through which features are inherited through generations.

In virtue of the three features above, epigenetics provides a chain of connections between what used to be qualified as *social* and *natural* inequality, leading to a reformulation of these contested boundaries. This also leads to a rethinking of the time-frame and scope of equality of opportunity.

## Epigenetics and Health Inequalities

We begin this review of the public health implications of epigenetics by situating a discussion of epigenetic mechanisms into the broader context of the social determinants of health inequalities.

The incidence of several diseases is negatively correlated with socioeconomic status (SES), as measured by income, wealth and educational level and independently from the universal public provision of health services. This striking epidemiological phenomenon is illustrated by the city of Glasgow, UK, where people in the poorest neighborhoods expect to live 12 years less than their counterparts in the richest parts of the city. Poverty explains only part of these disparities: a steady gradient of health outcomes across social classes has been observed for many conditions, even among groups that are fully above the threshold of poverty (Marmot, 2005).

Neither the steepness of the health gradient nor the magnitude of these disparities can be accounted for by the social stratification of a single kind of risk factor. Rather, material (e.g. poverty), behavioral (e.g. diet), biological (e.g. blood pressure) and psychosocial (e.g. stress) pathways may add up and interact to establish social health inequalities (Arendt and Lauridsen, 2007; Blane, 2006).

In our review we focus on the early life effects of SES. In ‘DOHaD and epigenetics’ section, we introduce the concept of ‘DOHaD’, the role of epigenetics as a mechanism for DOHaD, and for the inheritance of acquired predispositions (in a quasi-Lamarkian fashion). In ‘Case-studies’ section, we discuss two specific cases in more detail: nutrition and parenting style, associated, respectively, with metabolic diseases and psychological conditions. In section ‘Social epigenetics’, we provide some evidence of the social stratification of epigenetic differences between individuals.

### DOHaD and epigenetics

The concept of ‘DOHAD’ has been introduced to describe exogenous influences on early development that may predispose the organism toward specific phenotypes or diseases (Barker, 1995). Which is the mechanistic base of developmental programing? Epigenetics, the study of mitotically (or meiotically) heritable changes that alter gene expression and phenotypes, but are independent from the underlying DNA sequence, can provide some answer to this important question (Gluckman *et al.,* 2008). Epigenetics is the study of *semi-stable* molecular states (e.g. life long and transmissible over a limited number of generations) that influence physiology in subtle ways during development, in physiological conditions and in the establishment of several diseases. Also, they are *sensitive to environmental clues* rather than being exclusively endogenously determined and provide biological systems with flexibility, the capability to respond to environmental challenge and rudimentary ‘learning’ mechanisms. Both stability and sensitivity to the environment are features that render epigenetics an eligible mechanistic explanation of early programing.

Indeed it is this *semi-stability* or *relative stability* to confer such a pivotal role to epigenetics in a revised understanding of *natural traits* and response to environment. Indeed, epigenetic biological phenomena are slower than continuous dynamic gene expression changes affecting cells and organisms in their daily lives, yet faster than genetic inheritance and fitness, which occur at a slow pace over a large number of generations. At the same time they are able to respond to sustained environmental stimuli, and can be inherited through several, but possibly not many, generations. These characteristics taken together place epigenetics in a unique position in reshaping our understanding of how natural traits, traditionally viewed as heritable traits selected through countless generations during evolution, might actually evolve in a more dynamic manner as a response to environment, and thus, in the case of humans, to social environments.

The paradigmatic epigenetic phenomenon, in fact one that has been discovered early on, is DNA-methylation, the addition of a methyl group to a DNA cytosine residue, initially thought to occur mostly at cytosine-guanosine dinucleotides (CpGs) in promoter regions of genes, but now understood to be a more widespread phenomenon occurring throughout the genome also at non-CpG nucleotides (in embryonic stem cells) and away from promoters (in intergenic regions). Changes in the DNA-methylation patterns at the genomic level are involved in the regulatory control of gene expression in mammalian development and in the development of several human physiological traits as well as diseases. This epigenetic modification has its own control machinery, a set of enzymes (DNA methyl transferases), which generate *de novo* methylation patterns, as well as controlling the maintenance of DNA-methylation patterns throughout development. Although in mammals DNA-methylation is *reprogramed* in the earliest phases of development (first days after conception around the time of *in utero* implantation of the embryo), there is evidence of trans-generational persistence of DNA-methylation signatures (i.e. imprinting), which are also involved in the development of certain developmental conditions.

Recent advances in sequencing techniques (i.e. next-generation sequencing) rendered possible the high-throughput screening of genome-wide DNA-methylation patterns, thus providing the first genome-wide maps that will allow us to correlate molecular differences at the epigenetic level and significant health parameters, such as the susceptibility to cardiovascular diseases (CVDs). Thanks to next-generation sequencing, we are now also able to explore in a more complete manner other types of epigenetic signatures beyond DNA-methylation, i.e. those based on histone modifications (histone methylation, acetylation, etc). The number of modifiable histones, and the number of modifications observed so far have brought the scientific community to point to a ‘histone code’ because of its potential complexity. Nonetheless histone modifications have already been shown to be involved in epigenetic inheritance and epigenetic control of gene expression.

In conclusion, epigenetic mechanisms appear to be involved in the early programing of adult phenotypes and the transmission of molecular phenotypes across generations. In the next section, we will discuss the empirical evidence bearing on two classes of cases: metabolic disorders and psychological conditions.

### Case-Studies

#### Maternal diet and adult outset diseases predispositions

A paradigmatic case of the relevance of epigenetic programing for adulthood diseases is nutrition during pregnancy (Sullivan *et al.,* 2011). Subtler features of diets may program the newborn for conditions that will be visible only in adulthood. Both undernourishment and hypernutrition have been proposed to be influences of this kind. As for the former, in the studies of the population affected by the 1944–45 Dutch famine maternal undernourishment was shown to be a predictor of susceptibility to type II diabetes. Based on this, Neel (1962) proposed that maternal malnutrition may act as an environmental switch that turns the metabolic system of the developing fetus in a *thrifty* mode, which is adaptive if food scarcity is endemic, but becomes ‘*maladaptive*’ when it matches poorly an environment where there is plenty of high-energy foods. Similarly, a high-fat maternal diet programs her offspring for increased risk of adult obesity or metabolic disorders in childhood (Chmurzynska, 2010). Both these mechanisms may be relevant for the early-programing hypothesis in life-course social epidemiology, in fact in two different contexts of sharp disparities in health outcomes. The association between early undernourishment and metabolic disorders may explain the sharp increase in the prevalence of the latter in emerging economies, which in fact experience a ‘double-burden’ of diet-related conditions (Popki, 2001). The association between maternal hypernutrition and children conditions is instead important in high-income countries, where obesity is socially stratified and especially so among women: aside from behavioral channels of transmission of childhood obesity, which is predicted by parental body max index, programing may be partially responsible of this ‘inheritance’ of the condition (Sullivan *et al.*, 2011). Epidemiological studies have also begun indicating intergenerational transmission of DOHAD effects (i.e. observing a grandfather to grandson DOHAD effect) (Kaati *et al.*, 2007). These have been investigated in greater depth and across more generations in animal models. A study of a low-protein diet in rats, for example, has indicated that the phenotypic effects of the diet given to the so-called F0 generation (i.e. the first set of parents from which the experiment starts) are indeed observed not only in the F1 (i.e. in their children), but also in the F2 (i.e. their grandchildren) and finally the phenotype is lost in the F3, underlining the semi-stable heritable behavior of this programing effect (Harrison and Evans, 2009). Moreover, it was shown that the effects on hypertension of low-protein diet during fetal life were reversed on administration of pharmacological compounds blocking maternal glucocorticoid synthesis (Langley-Evans, 1997). Both the human cohorts and animal models have been investigated in terms of the potential epigenetic mechanisms involved in the DOHAD effect providing early clues to the role of DNA-methylation (reviewed in Mathers *et al.,* 2010).

#### Parenting style and psychological conditions

Health is important in its own right as for life opportunities. Also, poor health is associated with lower educational attainments and therefore health is partly responsible for the association between being born in a poorer family and lower educational achievements (Case *et al.*, 2005). The latter are a crucial predictor of employment status and income, and therefore health does impinge on opportunities in at least two ways: directly and through educational careers.

Moreover, there is a broader class of conditions that are certainly relevant for life opportunities and for which epigenetic mechanisms might be responsible as well: psychological conditions. In particular, stress responsivity, cognitive ability and response to reward are highly sensible to early-life events, especially maternal care (Champagne, 2008). Because these are also features that will influence opportunities, recent discoveries on the biology of early life circumstances and psychological conditions might open up new policy avenues to tackle disadvantages.

This DOHAD effect has also been furthered by experiments with animal models. A study on rats’ mothering styles gave some hints about how this might work (Weaver *et al.,* 2004): the stress response mechanism is tuned by maternal behavior in early life and it also leads to permanent changes in DNA-methylation and histone acetylation of genes that are involved in stress response. The main idea beyond the discovery is that maternal parenting style changes profoundly the physiology of the rat pups, triggering a cascade of molecular events that get written on their epigenetic code thus modulating gene expression in adulthood. The researchers studied a phenotype of mothering behavior, frequent licking and grooming (high-LG), that was associated with high level of hippocampal glucocorticoid receptor activity, which is related to stress response.

Because upregulated stress response is in turn associated with high-LG mothering style in adulthood, the study provided the description of an epigenetic trans-generational inheritance of a behavioral trait (Sapolsky, 2004). For our purposes (see section ‘Reversible and preventable epigenetic predispositions: correcting early disadvantage as investment’) it is, however, pivotal that these epigenetic changes might be amenable to modifications. Adoption of pups of high-LG rats by mothers displaying the low-LG phenotype restore the normal stress response phenotype (Darnaudéry *et al.*, 2004). More recently, the maternal programing of stress response was shown to be reversible on administration of DNA-methylation modulator drugs (Weaver *et al.*, 2005). Although these results were so far obtained in animal models, it should be clear how these findings might be relevant for researches who try to address the environmental insults in early infancy.

In conclusion, the epidemiological evidence of early programing and intergenerational transmission of metabolic and psychological conditions in humans is partly explained by epigenetic mechanisms, evidence of which is strong in animal models. Through epigenetic mechanisms, environmental insults in early infancy may program future metabolic and psychological phenotypes later in life (DOHaD); moreover, environmental insults experienced by parents and grandfathers can be transmitted to children and grandchildren (inter-generational inheritance).

Evidence of direct inheritance is still sparse and open to empirical scrutiny (Daxinger and Whitelaw, 2010). On the other hand, early programing could represent an *indirect* way in which health disadvantages are acquired and passed on from parents to children. This inter-generational stability of certain conditions is already known in social epidemiology at the statistical level. Behavioral and socio-structural channels of inheritance may account for this stability: children from poor backgrounds encounter social environments that are similar to their parents’ and *learning* is a major pathway of inheritance of unhealthy lifestyles. Jablonka and Lamb (2005) explicitly juxtaposed epigenetics and ‘cultural’ inheritance, fleshing out an analogy based on their inter- and intra-generational stability and their sensitivity to exogenous clues. Their ideas include the hypothesis that there could be more channels over and above cultural inheritance (e.g. learning) and genetics that explain how traits are inherited from parents to their offspring: in particular, epigenetics mechanisms would be sensitive to environmental clues, similarly to what happens in the case of learning, while being at the same time relatively stable through time, as in the case of genetics. Their hypothesis replaces the distinction between innate biological traits and flexible learned traits with a continuum of biological traits that are more or less programable by environmental clues and reprogramable by *post hoc* interventions. Epigenetics is thus a *sui generis* channel of inheritance, which shares features both with genetics (i.e. stability) and cultural transmission (i.e. learning processes).

### Social Epigenetics

McGuinness *et al.* (2012) reported the association between SES and global DNA-methylation in the pSoBid cohort, a study group with strong social health gradients from the city of Glasgow. Specifically, they found that vast hypomethylation was associated with severe deprivation and being a manual worker. Also, they observed a positive trend between number of years spent in education and global DNA-methylation. They independently tested the association of DNA-methylation with CVDs and inflammation markers, finding that CVD risk is associated with hypomethylation when controlling for SES and lifestyle factors. Borghol *et al.* (2012) have investigated this question in further detail, by identifying specific epigenetic markers linked to SES in the 1954 British Birth Cohort, which were found to cluster in specific regions of the genome linked to specific human functions (e.g. higher methylation, and thus repression, of sensory perception of smell and taste in low SES individuals).

In the next chapter, we will review some normative considerations that have been put forward to explain if, why and to what extent social inequalities are *unjust* and discuss whether the three features of social epigenetic traits induce any change in that ethical assessment of existing inequalities *and* the interpretation of the normative principles themselves. In fact, social epigenetic traits are morally important because they may strengthen existing normative considerations (or suggest new moral issues) but also because they cut across traditional distinctions that have been used to *express* the normative principles themselves, as the difference between acquired and innate features or between social and natural traits.

## Health Inequalities and Justice

A wide range of different, often irreconcilable, moral views supports the view that health inequalities are important for justice. Each view may provide a different explanation why health inequality, in itself, or when associated to social inequality, is unjust. In this article, however, we shall focus on the relationship between health and equality of opportunity and on the relationship between health and socially created inequalities.^1^ The idea of equality of opportunity is interpreted differently by different moral theories, concerning the relevance for equality of opportunity of natural vs. social inequalities.

So-called ‘luck’-egalitarians aim to equalize outcomes due to natural inequalities. They hold that it is unfair to suffer disadvantage from factors beyond one’s control, natural and social circumstances alike. Another version of the ‘luck-egalitarian’ idea invokes a ‘prioritarian’, rather than an ‘egalitarian’, rationale. According to prioritarianism, ‘one ought as a matter of justice to aid the unfortunate, and the more badly off someone is, the more urgent is the moral imperative to aid’ (Arneson 2000: 343). Thus, according to a *prioritarian* version of luck-egalitarianism (or equivalently, a ‘responsibility-catering’ version of prioritarianism), ‘the moral value of altering a state of affairs in a way that makes someone better off or worse off depends, other things being equal, on the degree of responsibility the person bears for her present condition’ (Arneson 2000: 344). Responsibility-catering prioritarians attach moral weight to redressing disadvantage for which people are not responsible, but they also attach moral weight to aiding the worse off, the more worse off they are. They judge social policy in terms of its aggregate utility outcome, but unlike traditional utilitarians, they attach more weight to generating utility for worse off people and if the disadvantage is due to bad luck.

For luck-egalitarians of both types, there is no intrinsic moral difference between a disadvantage that is a deliberate or accidental effect of social institutions and one produced by the genetic lottery. In education, for instance, because no one is alleged to deserve innate natural talents, luck-egalitarians might favor investing more resources in the education of the *least*-talented students, to close any sort of innate (e.g. genetic) gap. But luck-egalitarians may also allow investing more resources for the education of the most talented when it maximizes the return of the investment, neutralizing the influence of arbitrary factors on outcomes *ex post,* i.e. through *taxation* and *redistribution*: after redistribution, morally arbitrary factors (e.g. unequal talents at birth) ought not to engender unequal attainments in income terms (Hild and Voorhoeve, 2004).

Notice, however, that income compensation is only second best from the viewpoint of the people affected by bad brute luck. It places its recipients in a passive role, encouraging a pitiful attitude by others (Anderson, 1999). It can hardly offset the lack of social prestige, or the intrinsic self-realization rewards (Rawls, 1999; Taylor, 2004), attached to desirable social positions. Moreover, unequal power and responsibility is a probable cause of the social gradient of illness and disease, independently of income (Brunner and Marmot 2006). For this reason, it might be argued that the first best solution should be correcting the source of opportunity disadvantage at its outset, even by intervening on the distribution of natural talents, when feasible, through genetic technology and enhancement (Hunter, 2012: 42; Segall, 2010).

By contrast, Rawlsian egalitarians think that equality of opportunity requires removing social sources of disadvantage. The ‘Fair Equality of Opportunity Principle’ (henceforth FEO) requires the following:

assuming that there is a distribution of natural assets, those who are at the same level of talent and ability, and have the same willingness to use them, should have the same prospects of success regardless of their initial place in the social system. […] The expectations of those with the same abilities and aspirations should not be affected by their social class (Rawls, 1999: 63).

Notice that FEO does not require equal life chances for people whose *natural endowments* are not the same. This, one may argue, limits societal responsibilities to correcting disadvantage due to poor access to education, training, social networks and other *social* advantages. Biological (dis)advantage should be classified as ‘unequal natural assets’ and fall outside the scope of the principle. The difference between the luck-egalitarian and the Rawlsian conception of equality of opportunity is, thus, that the luck-egalitarian aims at maximally reducing the influence of morally arbitrary disadvantage, natural and social alike, while the Rawlsian aims at neutralizing the impact of social background.

In what follows, we discuss the relevance of DOHAD and epigenetics to these two conceptions of equality of opportunity.

### Implications of Epigenetics for Luck-Egalitarianism

What are the implications of DOHAD and epigenetics for the luck-egalitarian way of considering equality of opportunity? We shall consider two possibilities. In some cases, epigenetic traits may not be amenable to modification, at least given the present level of biomedical technological development. In others, they may be amenable to modification, but the development of therapies may require public investment.

#### Epigenetics as a risk monitor

In this section, we discuss the luck-egalitarian implications of discovering epigenetic predispositions not amenable to modification. As shown in section ‘Maternal diet and adult outset diseases predispositions’, some disease risks are programed since conception due to facts concerning the maternal environment. Clearly no one can be considered responsible for disadvantage accrued in this way. According to the luck-egalitarian conception of equality of opportunity, justice requires compensation of brute luck disadvantage, e.g. prenatally advantaged people ought to subsidize the health care costs of prenatally disadvantaged ones.

However, it is known that different individuals react to environmental insults to varying degrees (due to their genetic and physiological differences). Epigenetics might provide a measurable magnitude of the extent to which environmental insults have, indeed, caused harm in a person’s genome and thus cause predisposition to specific diseases, providing more accurate measures of disease risk due to environmental exposures. Epigenetic markers might thus become ‘health monitoring markers’, which provide an overall picture of accumulated environmental insult and epigenetic risk of disease. Imagine a society in which people can be informed by their family physician of the accumulation of risk factors due to specific environmental insults, including those arising prenatally and in early childhood for which people cannot be held responsible.

There are at least two important ethical consequences of this gain of information. First, epigenetic monitors might counterbalance currently skeptical views held by the public of environmental risk, whereby individuals heavily exposed to risk do not develop disease and constitute often cited exceptions to the statistical rule (e.g. ‘he/she smoked 40 cigarettes per day and yet did not develop lung cancer’). Consider a statistical risk factor, such as ‘watching TV for 3 hours a day in childhood leads to greater asthma risk’ (Sherriff *et al.*, 2009). Persuasion by statistical evidence will be greater if, amongst the population at risk (e.g. ‘children watching TV more than 3 hours a day’), epigenetics might aid in identifying the population more likely to be affected by the environmental risk (‘children watching TV more than 3 hours a day with early epigenetic evidence of increased asthma risk’) as opposed to the overall population exposed. We have known for a long time that many diseases do not strike blindly, but follow from a life-long accumulation of environmental insults, and that people from disadvantaged social backgrounds are more likely to experience unfavorable environments in their lifecourse too. But more precise epigenetic markers may help convincing the public of the importance of these factors.

The second is the possibility of direct applications. If in the future the epigenetic stratification of the disease, within the same environmental risk group, will be possible with a high degree of accuracy, it would develop a novel notion of ‘personalized risk’, whereby individuals who are less affected by certain risks (e.g. watching TV), could indulge more in those risks without the potential guilt associated with those behaviors in the general population and without the negative health outcomes. Moreover, the luck-egalitarian rationale of equality of opportunity justifies directing more resources for disease prevention to individuals who are at higher personalized risk. This kind of prioritization seems justified based on a luck-egalitarian rationale, as among people making similar choices (watching TV more than 3 hours a day), some people are more unlucky than others, due to physiological (e.g. genetic) and environmental factors (e.g. disadvantaged social background) beyond their control.

Notice that such information need not be made available to the public in ways that threaten personal privacy and might expose the victims of bad natural luck to further threats of discrimination. Access and utilization of sensitive medical records should be designed to maximize the privacy and reduce the threat of discrimination against adult citizens. A properly designed system may empower citizens to use morally relevant epigenetic information to their own advantage (e.g. justify health care entitlements for people in high-risk categories), while reducing their own privacy risks. People may voluntarily undergo an array of tests and then be granted some priority relative to health prevention and care. In contrast to what private health insurance coverage would do, governments would be justified, for instance, in subsidizing the adult patient with asthma who, as a child, was in the highest risk group, for no fault of his or her own. This would single out interventions targeting specific groups, e.g. parentally neglected children with special physiological vulnerability to environmental effects, as having some degree of priority over lower risk groups.

The availability of reliable indicators of early-life disadvantage supports the duty to contribute to the health care of others as a matter of equality of opportunity, understood along luck-egalitarian lines. But the point could be made that testing negative does not equate straightforwardly with being responsible of increased health needs. Other bad circumstances could get wired in the body in a yet unknown or undetectable fashion. Even the most sophisticated predictive tools, including epigenetic and nonepigenetic indicators, only capture a limited amount of circumstances due to brute luck.

In response, the argument is not meant to weaken the health care entitlements of those who are not demonstrably responsible for their bad health. It may well be irrelevant to societies that recognize a societal duty to meet citizen health needs unconditionally. But consider a society in which good health care and effective prevention is only for the people who can afford it, e.g. through private insurance. Relative to this baseline, the above proposed policy reduces the amount of morally arbitrary disadvantages. While leaving many morally arbitrary inequalities untouched, it still produces an improvement from a luck-egalitarian point of view if it contributes to equalizing health outcomes.

Notice that we are imagining a public use of epigenetics to justify public policies aiming at improving health in the direction of health *equality*, or *priority for the worst off.* Someone may understand luck-egalitarianism as the claim that a more just society would be one in which individual differences of responsibility have a greater impact on the health inequalities there are: there ought to be *both less* differences in health between the equally prudent (or reckless) *and more* differences in health between the prudent and the reckless. This would indeed be the most plausible consequence of taking a certain ‘desert-based’ view of justice: justice is achieved when those who make the greatest sacrifices of enjoyment and fun to protect their health enjoy comparably better health than the rest. But this is not the way luck-egalitarianism is standardly understood. Luck-egalitarians favor *removing* inequalities for which individuals are *not* responsible, not *promoting* inequalities that reflect responsibility: they maintain that there ought to be less differences between the equally prudent (or reckless) but they are indifferent, or even favor minimizing inequality between the prudent and the reckless.^2^

Finally, one potential implication of epigenetic testing is that it might be able to capture inherited initial disadvantage deriving from environmental insults of earlier generations. If conclusive evidence is provided that social disadvantage is transmitted across generations through biological channels, more people will understand the importance of meeting health care obligations by restructuring social institutions more broadly than just health care. As emphasized by the literature on the social determinants of health, social reforms for social mobility and reducing social inequalities at birth might have a greater aggregate impact on health than improving individualized health care. Suppose that socioeconomic inequalities affect the parental epigenome (as suggested by the evidence for epigenetic social stratification discussed in ‘Social epigenetics’ section) and that inherited features give socially advantaged children a better start in life (as suggested by the evidence of inter-generational transmission discussed in ‘Maternal diet and adult outset diseases predispositions’ and ‘Parenting style and psychological conditions’ sections). If so, achieving starting-gate equality of opportunity requires tackling inequalities of *outcomes* affecting the parental epigenome in a heritable way.

In conclusion, knowledge of epigenetic mechanisms may increase our ability to achieve (luck-egalitarian) equality of opportunity, by unraveling the mechanism through which the health prospects of a population are affected by the unequal choices and circumstances of their parents. (If epigenetics does not play an independent causal role, it will provide at least a reliable monitor of the impact.) Later on it will be pointed out that, to the extent that socioeconomic institutions play a causal role, there will be an overlap, in practice, between the luck-egalitarian and Rawlsian conceptions of equality of opportunity concerning epigenetic disadvantage.

#### Reversible and preventable epigenetic predispositions: correcting early disadvantage as investment

Let us now turn to the hypothesis that epigenetic changes be reversible. Recent data from both *in vitro* and *in vivo* experiments show that early acquired epigenetic predispositions might be. As we saw in section ‘Case-studies’, adverse health effects of early programing in rats (both nutrition and mothering style) can be reversed. Not only is the epigenome a ‘biosensor of exposure and/or outcome’ (Relton and Davey Smith, 2012: 7) increasing our diagnostic abilities (in ways that are relevant for justice) but it also opens up new avenues for innovative preventive interventions. It might be possible to design environmental or pharmacological interventions for reverting the potential adult consequences of a particular mothering style at the molecular, cellular and physiological level. One implication of luck-egalitarianism is that there is a *prima face* duty of justice to intervene: the possibility of reverting programed traits, when epigenetic information is a reliable biosensor, might efficiently prevent a process of life-time accumulation of disadvantage that ends up in disease.

This duty is only *prima facie*, meaning that on a tight budget, resources may have to be diverted to meet more immediate priorities. But the importance for health justice of preventive measures should not be underestimated. True, health prevention competes for resources with care for immediate health needs. But it can often be both more efficient and just to prevent diseases, rather than cure them. In what follows, we shall present some considerations in support of the idea that acting on early epigenetic determinants of diseases could be desirable from the point of view of efficiency.

Epigenetic disadvantage may matter in virtue of its early onset. If one considers harm to a person in a life-course perspective, early disadvantage, even of milder quantity, may involve a large disutility in the long run. This is clear in the ‘lifetime accumulation’ paradigm, in which genetic, epigenetic and socioeconomic determinants of bad health reinforce each other, leading to a continuous exposition to several drivers of diseases and social exclusion. Any loss of realized ability due to adverse upbringing is considered to be a loss (in productivity force) and might even correlate with some social ‘bads’, such as socially expensive exclusion that society would necessarily have to tackle later on (Esping-Andersen, 2002; Heckman, 2008).

The idea of a crucial time window, in which early programing takes place, is a feature of social epigenetic phenomena, which is highly relevant in this respect: traditional instruments of investment on human capital might in fact arrive too late if the vulnerability to diseases is established very early on. An epidemiological study (Feinstein, 2003) illustrates the establishment of relative cognitive capabilities in UK children: these are highly associated with social status and independent from baseline native levels but also determined soon after birth and relatively unamenable to modification on school entrance. This is challenging for policy makers because to obtain the same results that were previously thought to be achievable through schooling, institutions must be designed that reach infants at a young age, and in fact, even the condition of the previous generation ought to be tackled (Heckman, 2008).

When early investment in human capital and health of the population promotes economic and cultural growth to a sufficient extent to repay the initial investment, preventive policies should be supported as an efficient, as well as fair, way to tackle disadvantage and disease in the population.

### Implications for Rawlsian Egalitarianism

The duty to develop and provide therapies and social policies to prevent the accumulation of epigenetic risk rests on the assumption that social justice requires correcting the influence of causes of disadvantage for which people are not responsible, natural and social alike. It might be objected that moderate egalitarians (e.g. Rawls) are not committed to intervene in the distribution of the epigenetic predispositions people are born with. It is only natural for the people favored by the natural (epi)genetic lottery to end up with better health prospects. Those who are born with the worst natural assets can at most expect society to design a tax system that redistributes wealth to their advantage. They certainly cannot expect society to subsidize expensive medical services for the sake of correcting their unequal life chances.

Against this objection, epigenetics forces us to reconsider what counts as the natural/social boundary of equality of opportunity. The paradigmatic social disadvantage, in Rawls, is class disadvantage, namely being born and growing, until maturity, in a family low in the hierarchy of income and power. Social disadvantage is corrected by ensuring that all citizens, no matter their initial place in the social system, can access to education and training that fits their natural predispositions. By contrast, the paradigmatic instance of natural disadvantage is the unequal distribution of genetic predispositions, regarded as the outcome of a ‘natural lottery’.

The metaphor of a ‘natural lottery’ is misleading in the case of epigenetic traits that record environmental impacts and are inter-generationally transmitted. Consider an unfavorable predisposition to a common disease that is (i) induced by childhood malnutrition (‘Maternal diet and adult outset diseases predispositions’ section) or maternal deprivation (‘Parenting style and psychological conditions’ section), (ii) highly correlated with parental social disadvantage, (iii) transmitted to the next generation. Grandchildren in a socially disadvantaged family could inherit from their socially disadvantaged parents and grandparents a greater risk of metabolic disorders (‘Maternal diet and adult outset diseases predispositions’ section), or stress responsitivity (‘Parenting style and psychological conditions’ section). These are both ‘natural endowments’ and ‘socially generated’ endowments, partly explained by social disadvantage produced by human institutions. Even if a society where FEO is implemented subsidizes early health care and education, it may leave *inherited* epigenetic disadvantage untouched.

On a restrictive interpretation of FEO, these inherited differences are natural endowments because they are innate. Hence, FEO does not require financing medical treatments or social policies that can revert them or prevent them from causing further disadvantage. But a different, more extensive interpretation of FEO is at least equally plausible. The biological disadvantage of the grandchild is produced by adverse social conditions experienced by parents and grandparents. So it can be described as an effect of the *starting position in society*, just as the paradigmatic form of opportunity inequality FEO is concerned with, namely unequal access to education. If required to promote equality of opportunity, a liberal state may legitimately attempt to correct epigenetically inherited disadvantage by subsidizing special interventions, just as it attempts to ‘level the playing field’ by subsidizing the education of the poor.

As pointed out in section ‘Reversible and preventable epigenetic predispositions: correcting early disadvantage as investment’, this may take the form of designing pharmacological or lifestyle correctives for reverting programed phenotypes that are epigenetically measurable. But it is perhaps even more interesting to point out the consequences of the Rawlsian view, so interpreted, if epigenetic imprinting is not reversible. Paradoxically, epigenetic inheritance could entail that equality of opportunity can only be achieved by achieving a more equal distribution of outcomes. The ideal of equality of opportunity is usually put forward as an alternative to the ideal of equality of outcome because equality of opportunity (e.g. everyone having access to the same education or health care) is in theory compatible with outcome inequalities of any dimension. This assumption seems to be presupposed in the Rawlsian frameworks, where Fair Equality of Opportunity is distinct from (and constrains) the Difference Principle, a principle applying to the distribution of outcomes (income, wealth and the amount of power characteristic of every job and, more broadly, social position). The Difference Principle permits all inequalities of income, wealth and power that maximally benefit the worst off group in the population. Apparently, both Rawlsian equality of opportunity and the Difference Principle are compatible with large socioeconomic inequalities: Fair Equality of Opportunity allows all inequalities among the differently talented and motivated, while the Difference Principle justifies unequal rewards for different jobs (e.g. paying physicians and bank directors more than manual workers) when needed to attract more talented and motivated people to perform the most difficult and challenging jobs, if this contributes to improving the expectations of the least advantaged. Suppose that a change in workplace hierarchies, e.g. an increase of disciplinary powers for managers, improves the annual income of subordinate workers by 1000 €, while at the same time inducing in them a stress-related increase in smoking behavior and the consumption of fatty food. Let us also imagine that this produces epigenetic changes, which are passed to the next generation, so that at birth the children of subordinates are at greater risk of metabolic disease than the children of managers. In other words, there is a trade off between greater income expectations for generation N and more equal opportunities with respect to health for generation N + 1. In the Rawlsian theory, FEO constrains (being ‘lexically prior’ to) the application of the Difference Principle. Thus, when these circumstances obtain, the lexical priority of the FEO represents an objection against conferring more authority to managers to control subordinates, otherwise permitted by the Difference Principle. On the strict interpretation of FEO, this constraint is not justified: it does not affect the ‘expectations of those with the same abilities and aspirations’, but only the expectations of people born with different natural (epigenetic) endowments. On the broad interpretation, FEO commits society to ensure that the expectations of individuals ‘should not be affected by their social class’, *including when social class operates via natural endowments,* so it places limits to outcome inequalities in one generation for the sake of achieving more equal starting positions in the next.^3^

## Conclusions

Our discussion of luck-egalitarian and Rawlsian equality of opportunity in light of epigenetic discoveries leads us to three broad conclusions that might be relevant to public health:

1. Epigenetics can be considered a biomarker of brute-luck disadvantage: Epigenetic screening might diagnose early-life and inherited insults due to factors beyond personal control, thus giving people reasons to demand the provision of health services as a matter of equality of opportunity.
2. Reversibility of programed phenotypes: The adverse effects of inherited or early-life insults on health measurable through the epigenome might be reversible through early pharmacological therapies or environmental interventions, avoiding brute-luck health inequalities that are harder to tackle later on.
3. Nature vs. nurture: The social determination of epigenetic traits that can be inherited shows that innate traits are not necessarily insensitive to social structures. Reasons to tackle epigenetic disadvantage, being an instance of inherited social disadvantage, are also offered by the more restrictive Rawlsian version of equality of opportunity.

More generally, it makes a significant difference whether the natural inequalities mentioned by the Rawlsian theory are identified with different *genotypes* or different *epigenetic traits*. Epigenetic channels of inheritance challenge the received view on the distinction between social and biological inheritance of advantages and disadvantages. That view is based on what has been called *the DNA-centric donation/conception theory of inheritance of features* (Mameli, 2005): it explains biological similarities between parents and their offspring through the donation at conception of a privileged developmental material, DNA, while it leaves to cultural channels of transmission between parents and their offspring any other forms of similarity between them. The idea of the ‘natural lottery’ relies on that received view: traits that are explained by the donation of DNA at conception are due to the natural lottery, all the others are instead due to social structures. Epigenetics complicates the picture in two ways: (i) it expands the list of developmentally privileged material that can be passed on at conception from parents to their offspring (e.g. patterns of DNA-methylation); (ii) it suggests that biological inheritance does not happen only at conception by parental donation of developmental material: also environmental clues might program adult traits (i.e. early in development or in utero) and their intergenerational similarities, i.e. if parents and their offspring share the developmental environment; (iii) it suggests that biological inheritance is reversible through environmental clues and thus influences by social structure. If we are to retain a distinction between natural lottery and acquired traits we must explain how it may fit this updated picture of social and biological inheritance. Also, if we want to use that distinction in moral theory, we must explain why—if at all—the distinction between biologically and socially determined traits retains its moral weight when transferred in the new view suggested by DOHaD and epigenetics findings.

While it is important to bear in mind the distinction between a Rawlsian and a luck-egalitarian approach, on the issue of correcting for (social) epigenetic disadvantage a significant convergence between the two approaches obtains. While the social circumstances experienced by our parents and grandparents have no direct influence on the distribution of genotypes (save from mating patterns, with which, however, a liberal state cannot interfere), they may have a more direct influence on epigenetic predispositions. But if epigenetic traits are responsive to environmental cues, they can be influenced by social policy, and there is a fuzzy boundary between controversial ‘biopolitics’ (e.g. genetic enhancement) and traditional social policy focused on the main socioeconomic institutions (e.g. redistributive taxation, access to education) in the Rawlsian tradition. It is thus imperative from the point of view of public health and equality of opportunity to explore how the different ways of conceptualizing this boundary affect direction of scientific enquiry, ethical views and policy development.

## Conflict of Interest

None declared.

## Author Contributions

All authors contributed equally to the design and conceptualisation of this article. Michele Loi and Lorenzo del Savio wrote the sections ‘Introduction’ and ‘Health Inequalities and Justice’. Lorenzo del Savio and Elia Stupka wrote ‘Epigenetics and Health Inequalities’. All authors revised the whole manuscript and wrote the section ‘Conclusion’.

## Notes

1. Other influential theories start from an explicit explication of well being and a sufficentarian threshold of minimal welfare to point out that health inequalities signal coupled disadvantages that ought to be tackled or given priority by policy makers (see Powers and Faden, 2006; Wolff and de-Shalit, 2007).

2. Moreover, luck-egalitarians may emphasize the importance of external background factors on the choices that people make and will take a more nuanced position on diseases for which people are allegedly ‘responsible’ than their critics have assumed (Voigt, 2013).

3. Kollar and Loi (2013) discusses at a more abstract level Rawls's possible rationale for the reformulation of Fair Equality of Opportunity, using DOHAD and germ-line genetic enhancement as examples to justify the theoretical point. A similar argument concerning germ-line genetic enhancement is to be found in Loi (2011).
